# Surgical Management and Diagnostic Incisional Biopsy of Palisaded Neutrophilic Granulomatous Dermatitis (PNGD): A Case Report

**DOI:** 10.7759/cureus.115975

**Published:** 2026-09-08

**Authors:** Anhad Brar, Sai Erambalur, Vikram Vegesna, Christian C Sosa, Frederick Tiesenga

**Affiliations:** 1 Surgery, Windsor University School of Medicine, Cayon, KNA; 2 General Surgery, Community First Medical Center, Chicago, USA; 3 Surgery, All Saints University School of Medicine, Roseau, DMA; 4 Surgery, St George's University School of Medicine, St George's, GRD

**Keywords:** autoimmune skin disorders, diabetes and hospitalar hyperglycemia, infection control measures, palisaded neutrophilic and granulomatous dermatitis (pngd), skin lesion biopsy, small vessel vasculitis

## Abstract

This case report describes a 29-year-old previously healthy male patient who presented to the emergency room with a painful purpuric vesicular rash. The lesions started on the bilateral lower extremities and rapidly spread to his abdomen and bilateral upper extremities. Otherwise, he remained stable without systemic symptoms. The laboratory studies showed hyperglycemia (344 mg/dL) and elevated hemoglobin A1C (10.3%), revealing previously undiagnosed diabetes mellitus. Treatment started with empiric antimicrobial therapy and supportive care, along with a diagnostic skin biopsy performed in the operating room. He was discharged three days later with outpatient follow-up and glycemic counseling.

Our discussion explores the rare presentation of palisaded neutrophilic granulomatous dermatitis (PNGD) in a patient without a known autoimmune history. It highlights the role of a surgically obtained skin biopsy for a definitive diagnosis. In particular, this case links undiagnosed diabetes mellitus to the pathogenesis of PNGD. The report concludes that a systemic evaluation is key for granulomatous dermatoses, as managing underlying metabolic dysfunction can significantly improve the disease course and outcome.

## Introduction

Palisaded neutrophilic granulomatous dermatoses (PNGD) refer to a rare vasculitis that is believed to be a form of immune-complex-mediated vasculitis [1]. As such, it classically presents in patients with prior autoimmune disease [2]. However, as research into this disease increases, the scope of associated diseases continues to broaden. PNGD has been reported in association with diabetes, autoimmune hepatitis, Sjögren syndrome, Churg-Strauss, rheumatoid arthritis, systemic lupus erythematosus, systemic vasculitis, sarcoidosis, ankylosing spondylitis, and malignancies such as lung cancer [1-3]. However, given the wide range of inflammatory conditions, it is expected that the list would continue to grow.

Clinically, patients with PNGD most commonly present with centrally ulcerated lesions on extensor surfaces of the extremities with or without pain [3]. However, diagnosis requires both a clinically significant disease associated with increased inflammation and biopsy results. On biopsy, this granuloma is defined by the presence of macrophages and giant cells positioned around collagen degeneration [4]. Compared to other granulomatous dermatitides, there is a more intense accumulation of neutrophils and thus more degenerated collagen in the dermis [3]. 

Because of the broad range of associated diseases, it is important for medical practitioners to be aware of the possibility of vasculitis in their differential diagnosis of rashes and lesions. For example, in the case presented below, we shall discuss how a patient with PNGD was worked up, diagnosed, and treated.

## Case presentation

**Figure 1:**
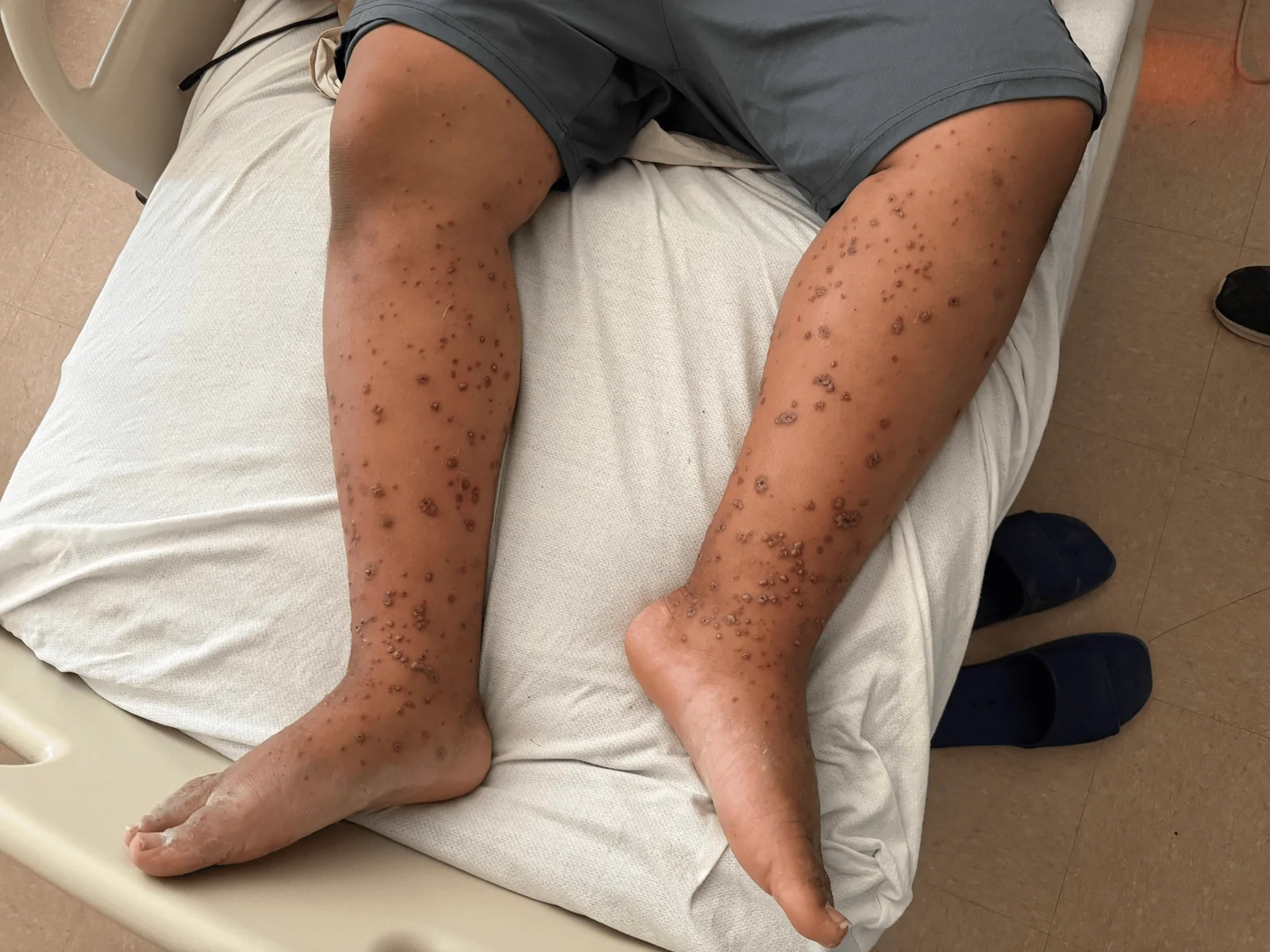
Initial presentation showing ulcerated papulovesicular diffuse rash of the bilateral lower extremities.

**Figure 2:**
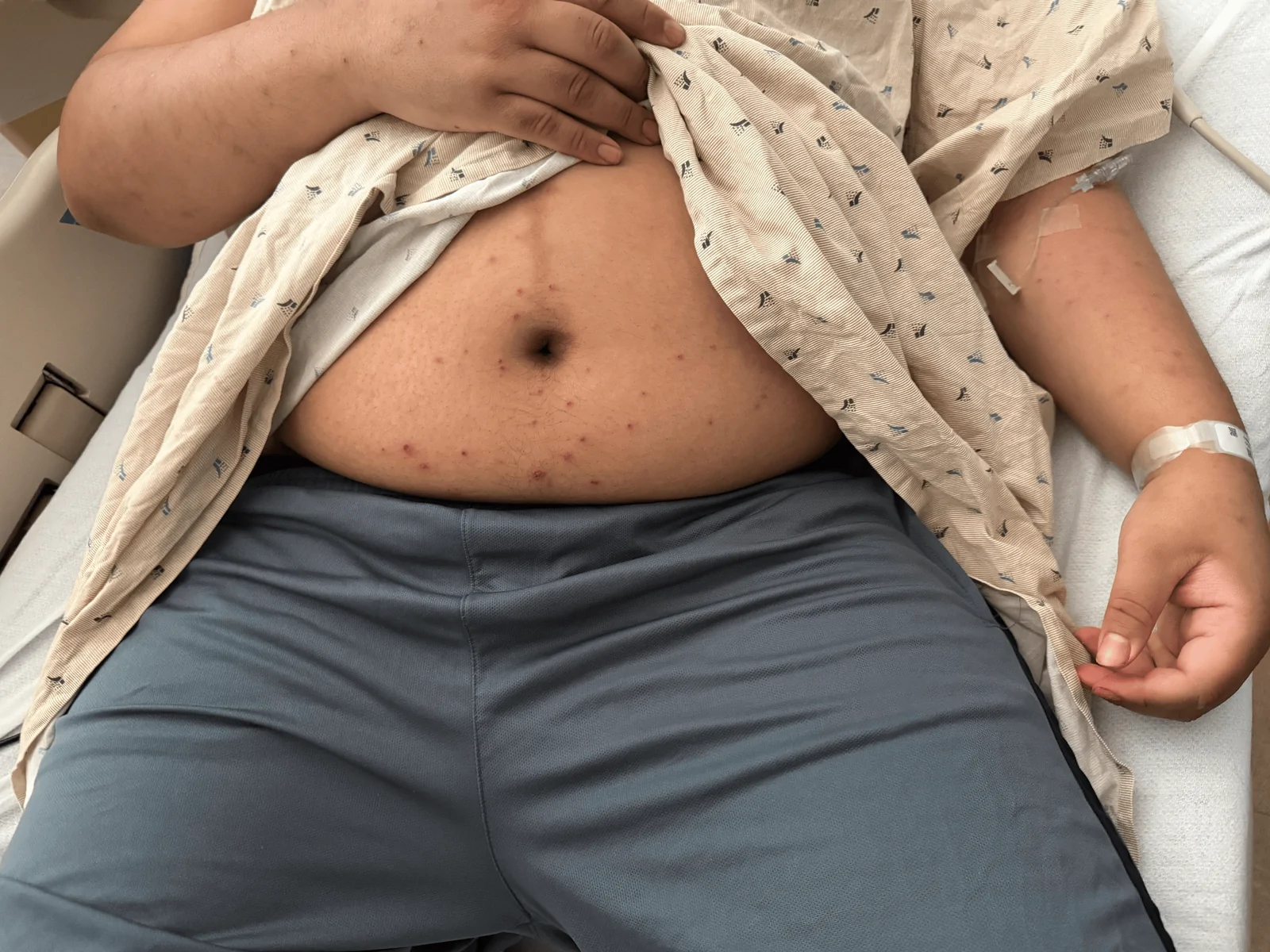
Eventual spread of the ulcerated papulovesicular rash to the lower abdomen and bilateral upper extremities.

A 29-year-old previously healthy man presented to the emergency department with a two-day history of a painful vesicular rash. The rash initially involved the distal aspects of both feet and ankles (Figure 1) and subsequently spread proximally to the thighs, inguinal creases, bilateral upper extremities, and abdomen (Figure 2) without involvement of the palms, soles, or genital region. The lesions were described as a burning sensation without pruritus. The patient’s mother did note that there were bed bugs in the home. The patient denied fevers, vision changes, systemic symptoms, recent travel, or known exposure to ticks or sick contacts. He reported occasional marijuana use but no other recreational drugs or alcohol. He was not immunocompromised and took no routine medications.

On the physical exam, vital signs were stable with the exception of BMI. BMI was noted to be 46.15. The rash was specifically observed as diffuse erythematous vesicular lesions with some areas of crusting on the lower extremities, abdomen, and bilateral upper extremities. Mild pedal edema was noted. The patient was alert, oriented, and in no acute distress. Cardiopulmonary, abdominal, and neurological examinations were unremarkable.

Laboratory evaluation revealed hyperglycemia with an elevated serum glucose and an elevated hemoglobin A1C (Table 1), indicating previously undiagnosed diabetes mellitus. Inflammatory markers ESR and CRP were mildly elevated (Table 1). HIV and syphilis screening were negative. Blood cultures, aerobic cultures with Gram stain, and rapid herpes/varicella zoster testing were pending at the time of initial evaluation.

**Table 1:** Quantitative laboratory investigations on admission. The patient's initial laboratory results demonstrated increased blood glucose, elevated hemoglobin A1c indicative of diabetes mellitus, and mildly elevated inflammatory markers (ESR and CRP).

Laboratory Parameter	Patient Value	Reference Range
Serum glucose (random)	344 mg/dL	< 140 mg/dL
Hemoglobin A1c (HbA1c)	10.3%	< 5.7%
Erythrocyte sedimentation rate (ESR)	69 mm/hr	0-15 mm/hr
C-reactive protein (CRP)	14 mg/L	< 10.0 mg/L

Infectious Disease and General Surgery were consulted. The patient was placed under airborne and contact precautions. One dose of insulin was given after blood glucose testing. Empiric therapy with valacyclovir and cefazolin was initiated.

A skin biopsy was performed for diagnostic clarification. The biopsy was interpreted with immunohistochemical (IHC) staining. Significant results were found only in the dermis. Specifically, the pathology team found a palisaded and interstitial neutrophilic granulomatous inflammatory cell infiltrate with focal collagen necrobiosis. PAS fungal staining was negative for fungi. Alcian blue mucin stain showed minimal mucin deposition.

**Figure 3:**
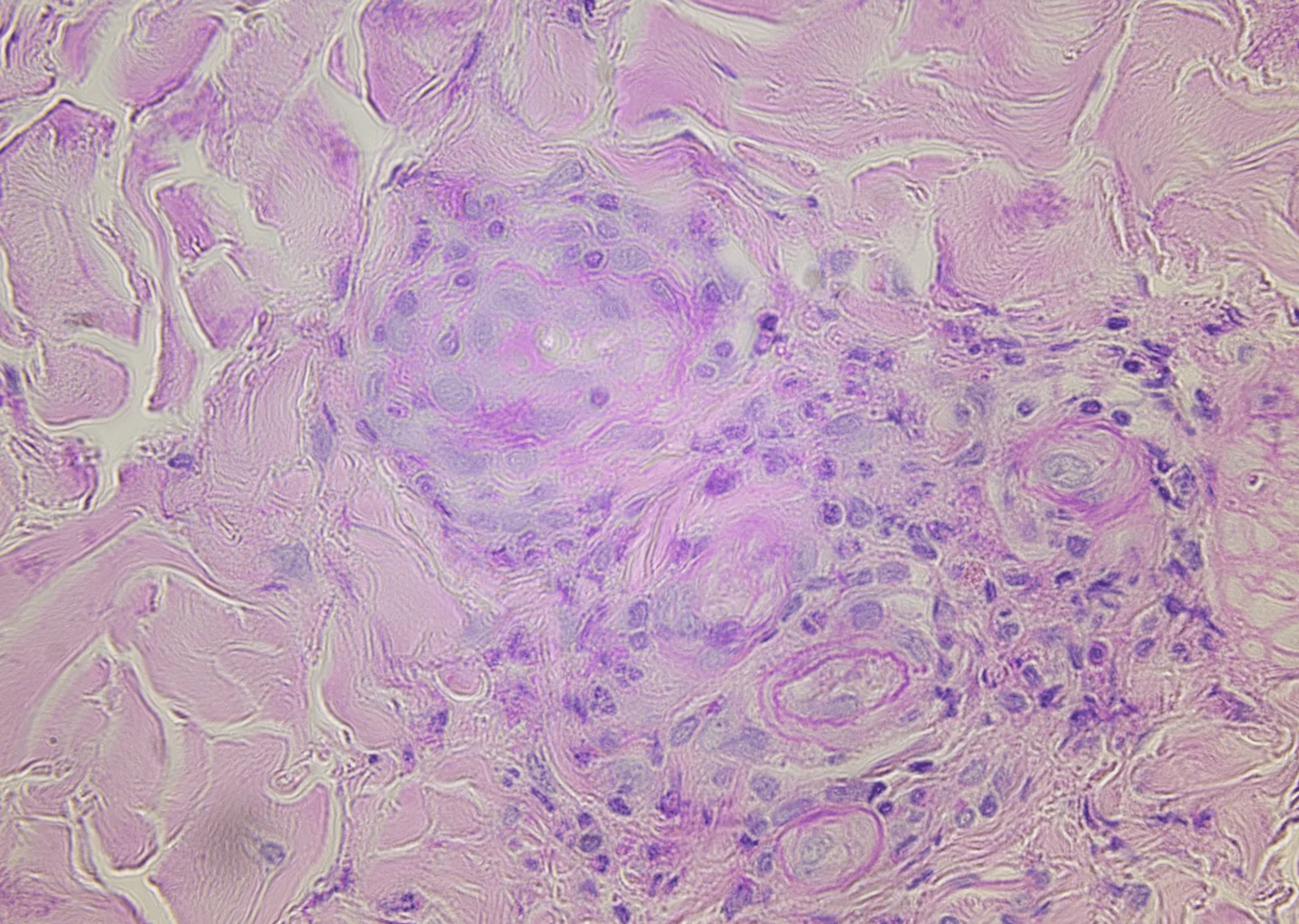
Immunohistochemistry stain with observed palisaded and interstitial neutrophilic granulomatous inflammatory cell infiltrates are observed with focal collagen necrobiosis. 40X oil immersion zoom.

Figure 3 demonstrates one of the observed palisaded and neutrophilic granulomas. Neutrophils are seen infiltrating and surrounding the formed granulomas in the observed dermis. The context of the observed neutrophilic and palisaded granuloma with the newly diagnosed diabetes in this patient comprises the two key pieces for the clinical diagnosis of PNGD.

Over the course of hospitalization, the patient remained hemodynamically stable, with no new systemic symptoms. Inflammatory markers and labs were trended, and the patient’s skin lesions were managed with supportive care. Because the patient was in stable condition per the Infectious Disease and Surgery team, the patient was cleared for discharge before the biopsy results could be thoroughly reviewed by the Pathology team. The patient was discharged home in stable condition on hospital day 3 with continued oral valacyclovir (1000 mg) and cephalexin (500 mg every 6 hours) for completion of therapy. He was counseled on glycemic control, infection precautions, and follow-up with primary care and infectious disease.

## Discussion

In our report, we discuss a rare case of PNGD in a 29-year-old male patient with no known history of autoimmune conditions. Physically, PNGD manifests as symmetrical red papules on the extensor surfaces of the elbows and fingers and may extend to non-classic locations such as the legs, cheeks, nose, and scalp. In our patient, they had such papules on their lower extremities and a few on the abdominal wall [5]. 

Prior to the diagnosis of PNGD, differential diagnoses that had to be considered were vasculitis, folliculitis, varicella-zoster virus reactivation, herpes simplex virus, arthropod infestation (bed bugs), or a bacterial skin infection. Because of this broad range of diseases, skin biopsy was necessary. What is most interesting is that PNGD or any granulomatous dermatoses were not considered as differential diagnoses. This fact highlights the rarity of PNGD as well as the importance of skin biopsy and clinical clues in being able to properly diagnose unique dermatological diseases. 

Early-stage biopsies demonstrate lymphohistiocytic cells with leukocytoclastic vasculitis and a pan-dermal infiltrate of neutrophils surrounding foci of degenerated collagen. Late-stage biopsies feature palisading granulomas, scattered leukocytes, fibrin, degenerated collagen fibers, neutrophil debris, and fibrosis [5]. 

Differential diagnosis includes leukocytoclastic vasculitis, rheumatoid nodules, granuloma annulare, Necrobiosis lipoidica, and interstitial granulomatous dermatitis (IGD). Compared with leukocytoclastic vasculitis, PNGD has extensive neutrophilic infiltration and severe collagen degeneration. Rheumatoid nodules are located deeper in the dermis and surrounded by granuloma. Granuloma annulare has less nuclear debris, thin degenerated collagen, and abundant mucin deposits. Necrobiosis lipoidica presents with a palisading granuloma with degenerated collagen extending through the dermis and subcutaneous fat [6]. 

The patient’s punch biopsy consisted of the epidermis and the dermis. The epidermis had unremarkable findings. Within the dermis, a palisaded and interstitial neutrophilic granulomatous inflammatory cell infiltrate was observed with focal collagen necrobiosis, showcasing late-stage lesions suggesting a histological overlap between PNGD and IGD. Its similar clinical and pathological nature sparks debate in the literature to classify the diseases as separate conditions or unified under the term reactive granulomatous dermatitis (RGD). IGD presents with symmetrical erythematous to violaceous plaques and patches on the upper trunk and/or proximal limbs. PNGD presents with plaques, papules, or nodules with central ulceration or crusting distributed on the extensor limbs. Ulcerated papulovesicular rashes have been observed at the upper and lower extremities and abdomen, suggesting the presentation is aligned with PNGD [7].

Its association as a clinical marker for a range of systemic conditions, infections, hematological malignancies, and medications such as antihypertensives, lipid-lowering agents, and anticonvulsants makes it crucial to ensure a proper diagnosis [8]. In our patient, he has not been taking any medications known to be associated with the development of IGD, such as antihypertensives, anticonvulsants, and lipid-lowering agents [6]. His unremarkable prior medical history, absence of systemic findings, newly found diabetes, and skin biopsy findings prove his condition to be PNGD. 

The pathogenesis of PNGD is not fully understood but is theorized to be due to deposition of immune complexes in the walls of the small dermal blood vessels, leading to a subacute or chronic neutrophilic-dominant small-vessel vasculitis. In turn, blood flow is impaired, causing degeneration of the surrounding collagen. This may incite an immune response, resulting in a palisading lymphohistocytic infiltrate. These events are likely precipitated by repeated trauma, which explains the clinical presentation on extensor surfaces [9].

We believe that in the context of this patient’s diabetes and histological findings, the patient likely had PNGD. As is the case with this patient (BMI of 46.15), obesity has been well documented to be associated with increased inflammation. Our understanding is that just as adipocytokines can upregulate inflammation in periadventitial tissue near the blood vessels, so too can adipokines stimulate an inflammatory response in obese patients [10,11]. Because of this heightened inflammatory state, it is logical to make an association between increased inflammatory markers and the possibility of developing autoimmune-associated pathologies. In addition, diabetes has been shown to be associated with PNGD in the past [3]. However, we recognize that diabetes was the only observable disease associated with PNGD at the time. We were unable to perform further rheumatological workup to exclude or affirm any other associated diseases because the patient was deemed to be stable before the Pathology team could interpret the biopsy results. Thus, we are unable to interpret long-term follow-up in the outpatient setting at this time. 

Treatment of PNGD is directed to the management of the underlying disease. Approximately 20% of patients experience spontaneous symptom resolution. Available treatments include non-steroidal anti-inflammatory drugs (NSAIDs), topical corticosteroids, prednisone, tacrolimus, and tumor necrosis factor (TNF) [8]. 

## Conclusions

PNGD is a rare dermatological entity associated with autoimmune disease and other inflammatory disease processes. This case highlights PNGD in a patient without a known autoimmune disease, with the notable finding of elevated blood glucose. The potential role of undiagnosed diabetes in the pathogenesis of PNGD remains speculative. In this case, a more extensive rheumatological and autoimmune workup could not be completed prior to discharge. Therefore, further reports of patients with PNGD, particularly those with metabolic abnormalities, may help clarify the pathogenesis and clinical associations of this condition.
